# Associations between the Intake of Different Types of Dairy and Cognitive Performance in Dutch Older Adults: The B-PROOF Study

**DOI:** 10.3390/nu12020468

**Published:** 2020-02-13

**Authors:** Liesbeth C. de Goeij, Ondine van de Rest, Edith J. M. Feskens, Lisette C. P. G. M. de Groot, Elske M. Brouwer-Brolsma

**Affiliations:** Division of Human Nutrition and Health, Wageningen University & Research, 6708WE Wageningen, The Netherlands; liesbethdegoeij@hotmail.com (L.C.d.G.); Ondine.vandeRest@wur.nl (O.v.d.R.); Edith.Feskens@wur.nl (E.J.M.F.); Lisette.deGroot@wur.nl (L.C.P.G.M.d.G.)

**Keywords:** dairy consumption, cognition, elderly, observational

## Abstract

Various dairy nutrients have been associated with cognitive performance. Several observational studies have explored associations between the intake of total dairy or some dairy subgroups and cognitive performance. However, studies on the potential impact of a broad variety of dairy subclasses are scarce. We examined cross-sectional associations between a wide assortment of dairy products and cognitive performance. A total of 619 Dutch community-dwelling adults aged ≥65 years completed a semi-quantitative Food Frequency Questionnaire. Cognitive performance was assessed with an extensive neuropsychological test battery; the tests were clustered into cognitive domains using z-scores. Linear and logistic regression analyses, adjusted for age, sex, BMI, education, smoking, alcohol consumption, habitual physical activity, total energy intake, and dietary factors, were performed to quantify the associations. The Benjamini–Hochberg method was used to correct for multiple testing. After full adjustment, higher skimmed dairy (β ± SD: 0.05 ± 0.02, *p* = 0.06), fermented dairy (0.04 ± 0.02, *p* = 0.09), and buttermilk (0.08 ± 0.03, *p* = 0.19) consumption were associated with better executive functioning. Logistic regression analyses indicated that a 30 g increase in Dutch cheese intake was associated with a 33% lower probability of poor information processing speed (PR = 0.67, 95% CI 0.47–0.97). No associations were observed between dairy consumption and attention and working memory or episodic memory.

## 1. Introduction

In the Netherlands, nearly 270,000 people have dementia, which is estimated to increase up to more than a half million by 2040 [1]. As there is, as yet, no effective treatment to cure dementia, strategies to prevent or slow down the development of cognitive decline are warranted [2,3].

Nutrition has been identified as one of the determinants of age-related cognitive decline [4,5,6], where beneficial associations have been shown for various dairy nutrients such as bioactive peptides, vitamin B_12_, calcium, and whey protein (reviewed in [7]). Associations between dairy consumption and cognitive decline may also be explained through the potential effects on cardiometabolic pathways [8,9]. Conversely, through adverse associations with cardiometabolic health^7^, specific fatty acids, sodium, and sugar—as part of e.g., full-fat dairy products, cheese, and sugar-sweetened dairy products—have been adversely associated with cognition [10]. Thus, certain dairy nutrients may reinforce, but also counteract each other’s effects. To facilitate the formulation of concrete recommendations, studies exploring the associations of specific dairy groups (e.g., skimmed, semi-skimmed, full-fat, fermented, non-fermented) or products (e.g., milk, yogurt, cheese) with cognitive performance are needed.

To date, only a limited number of observational studies and one meta-analysis have explored the association between dairy consumption and cognition [11,12,13,14,15]. Crichton et al. conducted a systematic review with meta-analyses involving three cross-sectional and five prospective studies [11]. Whereas the results of cross-sectional studies were inconsistent, four out of five prospective studies observed an inverse association between higher full-fat dairy consumption and poorer cognitive performance. Most studies assessed overall cognitive performance or decline with the Mini Mental State examination (MMSE); a tool that may not be sensitive enough to detect cognitive differences in relatively cognitively healthy populations. In addition, most studies only explored the potential impact of the consumption of total dairy or some dairy subgroups. 

Therefore, the aim of this study was to investigate associations between a broad variety of dairy subclasses and dairy products with domain-specific cognitive performance in Dutch adults aged ≥65 years.

## 2. Materials and Methods

### 2.1. Participants

This cross-sectional study was performed using baseline data of the B-PROOF study [16], a randomized, double-blind, placebo-controlled trial designed to assess the effect of daily oral supplementation of vitamin B_12_ (500 µg) and folic acid (400 µg) on fractures in 2919 mildly hyperhomocysteinemic adults ≥65 years. Participants were recruited at three study centres (i.e., Wageningen University, VU University, and Erasmus Medical Centre Rotterdam) from August 2008 to March 2011. Participants with homocysteine concentrations <12 µmol/L or >50 µmol/L, and creatinine concentrations >150 µmol/L were excluded. Only participants recruited in the surroundings of Wageningen (*n* = 856) underwent cognitive testing (*n* = 856) and completed a Food Frequency Questionnaire (FFQ) (*n* = 665). Of the 665 participants, 619 were available for analyses. Participants with potentially unreliable or incomplete FFQ data [i.e., men with energy intakes <800 kcal or >4200 kcal, women <500 kcal or >3500 kcal (*n* = 46)] were excluded [17]. The excluded and included baselines did not differ, except for age, which was slightly older in the excluded group (75 compared to 72 years old). Further details on the design and methods of the B-PROOF study can be obtained from the design article [16]. This study was conducted according to the guidelines laid down in the Declaration of Helsinki and all procedures involving human participants were approved by the Medical Ethics Committee of Wageningen UR (ABR 20783.081.07). Written informed consent was obtained from all participants. The B-PROOF study was registered with the Netherlands Trial Register (trialregister.nl) as NTR1333 and at clinicaltrials.gov as NCT00696414.

### 2.2. Dietary Assessment

A 190-item Food Frequency Questionnaire (FFQ) was administered to assess habitual dietary intake where the previous month served as the reference period. The questionnaire was filled in at the participant’s home. The FFQ was designed to cover 96% of the absolute level of food intake and 95% of the between-person variability of each nutrient under study, as assessed in the Dutch National Food Consumption Survey of 1998 [18]. Participants answered questions relating to frequency by selecting answers ranging from ‘never’ to ‘6–7 days per week’. Portion sizes were estimated using natural portions and commonly used household measures. Frequency and portion size of consumed foods were multiplied to obtain intakes in grams per day. Subsequently, the average daily nutrient intakes were calculated using the Dutch food composition table (NEVO) (2011) [19]. The FFQ included information on all major nutrients and food groups, and was validated for total energy, macronutrients, dietary fibre, and selected vitamins [20,21,22]. For this study, dairy products were clustered as total dairy, skimmed dairy, semi-skimmed dairy, full-fat dairy, non-fermented dairy, fermented dairy, total milk, total yoghurt, total cheese, Dutch cheese, chocolate milk, buttermilk, and custard. Additionally, milk and yoghurt were subdivided according to their fat content, and cheese was subdivided into total cheese and Dutch cheese (Table 1). Only products that were queried in sufficient detail and could be classified by fat content based on the Dutch Commodities Act Dairy could be classified based on fat content [23]. All dietary variables were adjusted for energy according to the residual method, as suggested by Willet and colleagues [24].

### 2.3. Cognitive Performance

Cognitive performance was assessed at baseline according to a standard protocol. The measurements were performed during a 1-h session at one of the study centres by well-trained staff. The tests were performed in a room with as few stimuli as possible. Only the interviewer and participant were present in this room. The participants practiced each test before it was administered. Global cognitive performance was assessed with the MMSE [25]. An extensive neuropsychological test battery was used to assess four cognitive domains. The domain attention and working memory was evaluated using the Digit Span forward and backward from the Wechsler Adult Intelligence Test (number of correct items) [26]. The domain executive functioning was evaluated using the Trail Making Test (TMT) part-B (seconds) [26], the Stroop Color-Word Test part-III (seconds) [27], and the Letter Fluency (number of correct items) test [28]. The domain information processing speed was assessed with the TMT part-A (seconds) [29], the Stroop Color-Word Test part-I and II (seconds) [27], and the Symbol Digit Modalities Test (SDMT) (number of correct items) [30]. To control for motor speed, interference measures were calculated for the TMT (TMT part-B/TMT part-A) and the Stroop Color-Word Test (Time needed Stroop part-III—[mean time needed for Stroop part-I and II]). The domain episodic memory was evaluated using the Rey Auditory Verbal Learning Test (number of correct words), which was used to assess immediate and delayed recall, as well as word list recognition [31]. For each domain, individual Z-scores were calculated resulting in compound scores for the different neuropsychological domains [32].

### 2.4. Biochemical Analyses

Blood samples were obtained from participants who came in the morning to the research centre or to external locations in the living area of participants. Participants were instructed to fast overnight, or to take only a light breakfast. Blood sampling was performed by a skilled nurse. The samples were stored at −80 °C until further analyses. The plasma glucose concentrations were analysed using a hexokinase method and insulin levels were determined using an immunometric assay. 

### 2.5. Covariates

Height and weight were measured at one of the study centres; body mass index (BMI) was calculated as weight/height^2^. Data on education level (primary, secondary, or higher education), smoking status (non-smoker, current smoker, former smoker), physical activity (kcal/d) [33], and alcohol consumption (light, moderate, excessive) [34] were collected by means of self-reported questionnaires which were filled in at the participant’s home.

### 2.6. Statistical Analyses

Participant characteristics were reported as mean with standard deviation (mean ± SD), or *n* with percentages (*n*, (%)). Medians with interquartile ranges (median (IQR)) were used to report skewed variables. Chi-square tests were performed to assess the differences in the categorical variables over tertiles of total dairy intake. Kruskal–Wallis tests were performed for skewed continuous variables and one-way ANOVA of normally distributed variables. Linear regression analyses, as well as logistic analyses, were conducted to quantify the associations between dairy intake and cognitive performance, resulting in either β’s (g/day) or odds ratios (ORs) (100 g/day, with the exception of cheese, which was analysed per 30 g/day), respectively. As the rare disease assumption applied to this study, calculated ORs can be interpreted as prevalence ratios (PR) [35]. PRs were calculated by tertiles of dairy intake or for consumers vs. non-consumers (if less than 300 consumers), which resulted in the probability of being a poor cognitive performer (i.e., belonging to the poorest 10% of the population). A population-based 10% cut-off point is commonly used in cognitive research [36] and has been shown to be predictive for cognitive impairment [37]. All analyses were adjusted for age, sex (Model 1), BMI, education, smoking, alcohol consumption, habitual physical activity (Model 2), and total energy intake (Model 3). Also, the impact of the dietary factors, including total energy intake, meat, fish, vegetables, fruit, and bread, were examined by individually adding these variables to Model 3. To explore potential mediation by markers of glucose homeostasis in the associations between dairy intake and domain-specific cognitive performance, plasma glucose and plasma insulin were added, independently, to the fully adjusted model (Model 4, data not shown in tables). The analyses were adjusted for multiple comparisons using Benjamini–Hochberg correction for a false discovery rate of 0.20 [38]. Adjusted *p*-values smaller than the false discovery rate (0.20) are significant. The analyses were performed by using IBM SPSS Statistics Version 22. 

## 3. Results

Population characteristics are shown in Table 2. Participants were on average ± SD 71.9 ± 5.1 years old, had a mean BMI of 27.1 ± 3.7 kg/m^2^, and a mean MMSE score of 28.4 ± 1.7. Sixty percent of this population was men, 34% completed higher education, and 59% were former smokers. The mean plasma glucose concentration was 5.8 ± 1.4 mmol/L and the median (IQR) insulin concentration was 65 (40–126) pmol/L. The median total dairy intake was 325 (201–428) g/day. The analyses over tertiles of total dairy intake indicated statistically significant differences for the intake of alcohol, fruit, bread, proteins, carbohydrates, fat, and fibre.

In Table 3 and Table 4, associations between dairy consumption and cognitive performance (domain z-scores) are presented, quantified using β’s and PRs. After full adjustment, no significant associations were observed between any of the dairy groups and attention working memory or episodic memory, or between non-fermented dairy products and any of the cognitive domains. Conversely, significant associations were observed between higher fermented dairy, skimmed milk and buttermilk intakes, and better executive functioning, β ± SD: 0.04 ± 0.02, *p* = 0.03, β ± SD: 0.05 ± 0.02, *p* = 0.01 and β ± SD: 0.08 ± 0.03, *p* = 0.02. Logistic regression analyses suggested a 43% non-significant lower probability of a poor performance for information processing speed for a 100 g increase in buttermilk intake (PR 0.57, 95% CI 0.31–1.06). Finally, associations were also observed for another fermented product, namely cheese, where a 30 g increase in Dutch cheese intake was associated with a 33% significant lower probability of a poor performance on the domain information processing speed (PR 0.67, 95% CI 0.47–0.97). For all associations, additional adjustment for plasma glucose or plasma insulin did not alter the results (data not shown).

## 4. Discussion

In this study, higher intake levels of fermented dairy, skimmed dairy, and buttermilk were associated with better executive functioning scores. Moreover, an association was observed between higher Dutch cheese intakes, and better information processing speed scores. No associations were observed between total dairy consumption, dairy product consumption classified by fat content, or non-fermented dairy consumption, and the four cognitive domains. 

To date, six cross-sectional [13,14,15,39,40,41] and four prospective studies [12,42,43,44] have investigated associations between dairy intake and cognition. Most studies were conducted in adults aged ≥55 years [12,14,39,40,41,42,43,44] and used a global dementia screening tool such as the MMSE [13,39,40,41,42]. Our null findings for total dairy consumption are in line with two previous studies [12,15], but in contrast to three other studies [13,14,39]. In the Maine-Syracuse Longitudinal Study (MSLS), participants regularly consuming dairy had a lower odds of having a low global cognitive performance (OR: 0.15, 95% CI: 0.07–0.34), visual-spatial memory (OR: 0.34, 95% CI: 0.15–0.77), working memory (OR:0.28, 95%: CI: 0.13–0.58), and executive function (OR:0.39, 95% CI: 0.18–0.85), compared to participants never/rarely consuming dairy [13]. In the NHANES study, dairy consumption was associated with higher story recall (49.6 ± 0.7 vs. 43.7 ± 1.3, *p* = 0.0001) and Digit Symbol Substitution Test (DSST) (51.5 ± 1.9 to 46.2 ± 3.0, *p* = 0.009) percentile scores [14]. In line with this, Lee and colleagues [39] observed a positive correlation between total dairy intake and cognitive performance in women (*r* = 0.173, *p* < 0.01).

In our study, analyses exploring the impact of dairy products subdivided by fat content (i.e., skimmed, semi-skimmed, and full-fat dairy) only showed a positive association between skimmed dairy consumption (of which 59% was buttermilk) and executive functioning. The results of the MSLS, where milk consumption was classified by fat content, do not suggest different associations with cognitive performance [13]. In contrast, the consumption of full-cream milk was associated with impaired cognitive function (OR = 0.59, 95% CI: 0.37–0.94) among older Australian men [42]. Among middle-aged South Australians, whole fat ice-cream consumption was inversely associated with memory (β = −0.13, *p* < 0.001) and whole fat cream was positively associated with cognitive failures (β = 0.09, *p* < 0.05) in men, whereas low fat yoghurt intake was positively associated with memory recall in men (β = 0.10, *p* = 0.03) [15]. 

We observed significant associations of total fermented dairy consumption and buttermilk with cognitive performance. Up to now, none of the observational studies have examined the association between fermented dairy and cognitive performance or decline. However, one randomized, double-blind and controlled trial, including 60 patients with Alzheimer’s disease, assessed the effects of supplementation of fermented milk with a mixture of probiotics for 12 weeks on cognitive performance and showed a significant improvement in the MMSE score compared to the control group supplemented with milk (−5.03 ± 3.00%, *p* < 0.001) [45].

As potential dairy product effects may be related to particular product-specific nutrients, we hypothesised that more detailed analyses on the product level could provide more insight into the potential link between dairy product intake and cognitive performance. With respect to the individual product groups, we can conclude that our null findings for milk are in line with five other studies [13,15,40,41,44]. Conversely, the SU.VI.MAX-2 study showed lower verbal memory scores among consumers with high milk intake compared to consumers with low milk intake (mean difference T3 vs. T1 = −0.99, (−1.83–−0.15)) [12]. Pertaining to cheese, we observed that an increase of 30 g in Dutch cheese consumption was associated with a 33% lower probability of having a poor information processing speed. In line with this, the NHANES study showed higher information processing scores (DSST scores 52.0 ± 0.8 vs. 48.4 ± 1.2, *p* = 0.02) and higher story recall scores (51.9 ± 0.9 vs. 47.3 ± 0.7, *p* =< 0.0001) among cheese consumers compared to non-consumers [14]. Moreover, higher cheese intake was associated with a reduced probability of cognitive impairment in the State-wide Survey of Alabama’s Elderly (OR = 0.68, 95% CI 0.47–0.99) [41]. Conversely, four other studies did not observe associations between total cheese intake and cognition [12,13,15,44]. Our null findings for yoghurt intake and cognitive performance are in line with three other studies [12,13,44], but in contrast to one study showing positive associations between total yoghurt (β = 0.12, *p* < 0.05) and low-fat yoghurt (β = 0.10, *p* = 0.03) with quality of memory recall in men [15]. Although there do not seem to be specific patterns in methodological characteristics explaining the different findings of the summarized studies, null-associations seem to be somewhat more common in studies using global dementia screening tools.

Up to now, research on the underlying mechanisms explaining associations between dairy consumption and cognitive performance is in its very early stages. In our study, total fermented dairy, buttermilk, and Dutch cheese, which are all fermented dairy products, were associated with cognitive performance. As experimental studies have suggested an important role for gut microbiota in the central nervous system, called the gut–brain axis [46], these associations may be explained by the probiotic effect of lactic acid bacteria [6]. More specifically, pre-clinical studies have indicated that probiotics attenuate pro-inflammatory cytokines, decrease oxidative stress, and increase brain-derived neurotrophic factor, and as such may promote neuronal growth and survival [6,47]. Probiotics have also been related to an increase in tryptophan, which enhances the synthesis of serotonin in the brain [6,48]. Furthermore, fermented dairy products are rich in vitamin K2, which tends to be relatively high in Dutch cheeses [49]. Evidence is increasing that vitamin K2 is positively associated with cognition. Vitamin K2 is involved in the synthesis of sphingolipids and the biological activation of vitamin K-dependent proteins. Insufficient levels may cause dysfunction in the CNS [50]. Lastly, bioactive peptides have been shown to exert various activities affecting the cardiovascular, digestive, immune, and nervous systems [51]. For instance, middle-aged Gouda cheese has been suggested to contain blood pressure-reducing peptides with potent angiotensin-converting enzyme inhibitory activity [6,51].

Finally, there are also some limitations and strengths of the present study that need to be discussed. First, as results are based on cross-sectional data, it is not possible to draw any conclusions regarding causality. Secondly, our participants had slightly elevated homocysteine concentrations and consequently the results may not be generalizable to general older populations. Thirdly, some individual dairy products were consumed by a relatively low number of participants, which may have attenuated potential associations. Furthermore, dairy intake and cognitive performance were not assessed at the exact same time. However, as the FFQ measures habitual food intake and older adults are assumed to have reasonably stable eating patterns, we do not expect a substantial impact on the results. Moreover, the FFQ did not distinguish between fermented and non-fermented cheeses. However, as most cheeses are fermented with exception of fresh cheeses, we also do not expect a major impact of this drawback. Furthermore, dairy consumption in our population was relatively high. It may be that participants with the lowest dairy intakes had such high dairy intakes, being either favourable or non-favourable, already reaching the plateau in detecting associations between even higher intakes and cognitive performance. Finally, the fact that the questionnaire was only completed by 665 of 856 participants may relate to the fact that FFQs are limited by various methodological factors and burdensome for participants. For instance, FFQs are long questionnaires that may take up to 90 min to complete [52]. Although we did not ask participants why FFQs were not returned, practical experience has taught us that this is a major factor. Moreover, FFQs rely on memory, i.e., in recalling types of foods eaten, frequency and portion sizes; difficulties in recalling diet may have been a reason for participants for not returning the FFQ as well. The strengths of this study include the use of an extensive cognitive test battery, the possibility to adjust for a large number of potentially relevant covariates, the possibility to study a broad variety of dairy subgroups and the use of the Benjamini–Hochberg method to correct for multiple testing. In fact, to the best of our knowledge, this is the first observational study examining the association between fermented dairy consumption and cognitive performance.

## 5. Conclusions

The findings of this study in Dutch older adults show some positive associations between the intake of fermented dairy and skimmed dairy, in particular buttermilk, and executive functioning and/or information processing speed. No associations were observed for attention working memory and episodic memory. To confirm our results, further studies are needed using cognitive measures with sufficient sensitivity, including neuropsychological test batteries and more advanced imaging techniques, and detailed information on habitual dairy intake.

## Figures and Tables

**Table 1:** Definition of dairy product groups.

Dairy Product Group	Dairy Intake (g/day)		Included Dairy Products *
	Median	IQR	
Classified by fat content			
Total dairy	325	201–428	All dairy products, except butter
Skimmed dairy	32	0–150	Skimmed milk (11%), skimmed yoghurt (30%), buttermilk (59%)
Semi-skimmed dairy	62	11–169	Semi-skimmed milk (61%), semi-skimmed yoghurt (34%), 20/30+ cheese (3%), 40+ cheese (2%)
Full-fat dairy	54	20–120	Full-fat milk (16%), Full-fat yoghurt (27%), custard (27%), 48+ cheese (5%), full-fat cheese (1%), milk-based ice cream (8%), cream (16%)
Total milk	30	0–149	Skimmed milk (13%), semi-skimmed milk (65%), full-fat milk (22%)
(Semi-)skimmed milk	7	0–150	Skimmed milk (13%), semi-skimmed milk (87%)
Full-fat milk	0	0–0	Full-fat milk
Total yoghurt	90	18–146	Skimmed yoghurt (34%), semi-skimmed yoghurt (33%), full-fat yoghurt (33%)
(Semi-)skimmed yoghurt	54	0–129	Skimmed yoghurt (52%), semi-skimmed yoghurt (48%)
Full-fat yoghurt	0	0–10	Full-fat yoghurt
Total cheese vs. Dutch cheese			
Total cheese	31	20–47	All types of cheese; Dutch cheese (66%), other cheese (34%),
Dutch cheese	20	13–34	20+/30+ cheese (26%), 40+ cheese (14%), 48+ cheese (60%)
Individual main groups			
Chocolate milk	0	0–13	All types of chocolate milk
Buttermilk	0	0–40	All types of buttermilk
Custard	0	0–28	Mousse (30%), full-fat custard (70%)
Fermented vs. non-fermented			
Fermented dairy	158	75–235	All types of yoghurt (57%), buttermilk (22%), cheese (21%) and quark (0.01%)
Non-fermented dairy	97	24–192	All types of milk (70%), chocolate milk (16%), milk-based ice cream (0.07%), cream (0.08%) and custard (14%)

* Proportions of included dairy products in the dairy groups are expressed as a percentage.

**Table 2:** Population characteristics, overall and by tertile dairy intake.

	Tertiles of Total Dairy Intake								
	Total Population		T1 ≤235 (g/day)		T2 236–374 (g/day)		T3 ≥375 (g/day)		p *
*n*	619		206		207		206		
Total dairy intake, g/day	325	201–428	162	90–201	325	273–348	482	428–546	<0.0001
Sex, men (%)	369	60	120	58	109	53	138	68	0.006
Age, y	71.9	5.1	72.3	5.5	71.5	4.8	72.1	5.0	0.25
BMI, kg/m^2^	27.1	3.7	27.2	3.9	27.2	3.7	27.0	3.6	0.87
Glucose, mmol/L ^†^	5.8	1.4	5.8	1.4	5.8	1.4	5.8	1.4	0.98
Insulin, pmol/L ^‡^	65	40–126	68	41–120	64	41–134	65	39–125	0.99
Smoking, *n* (%)									0.32
Non	189	30	55	27	70	33	64	31	
Current	65	11	28	13	18	9	19	9	
Former	365	59	123	60	119	58	123	60	
Physical activity (kcal/day)	599	362–875	578	367–858	636	373–941	605	354–854	0.41
Education, *n* (%)									0.38
Primary	261	42	93	45	77	38	91	44	
Secondary	148	24	42	20	59	28	47	23	
Higher	210	34	71	35	71	34	68	33	
Alcohol intake, *n* (%)									0.002
Light	397	64	127	62	120	58	150	73	
Moderate	204	33	68	33	84	41	52	26	
Excessive	18	3	11	5	3	1	4	1	
MMSE (range 0–30) ^§^	28.4	1.7	28.3	1.8	28.4	1.6	28.3	1.6	0.76
Energy, kcal/day	1948.1	497.3	1967.3	485.9	1913.6	486.3	1963.3	519.6	0.47
Protein, En%	15	5	13	4	16	4	17	4	<0.0001
Carbohydrates, En%	45	6	39	6	45	5	50	5	<0.0001
Total fat, En%	36	7	32	6	36	7	39	7	<0.0001
Fibre, g/day	24	6	22	6	24	6	26	7	<0.0001
Meat, g/day	73	49–97	73	47–94	73	53–95	74	47–100	0.83
Fruits, g/day	172	92–254	149	73–236	174	92–259	193	109–267	0.003
Fish, g/day	11	4–18	10	4–18	11	4–20	11	4–17	0.74
Bread, g/day	112	79–148	106	76–142	112	80–150	116	83–154	0.02
Vegetables, g/day	118	81–166	111	72–142	117	84–160	121	86–177	0.10

Data are expressed as mean (SD), median (IQR) or *n* (%). * Chi-squared tests for categorical variables, Kruskal–Wallis for medians of continuous variables and one-way ANOVA for means of continuous variables. ^†^ Data available for *n* = 610. ^‡^ Data available for *n* = 609. ^§^ Data available for *n* = 617.

**Table 3:** Associations between intake of dairy intake groups (g/d) and cognitive performance (domain *z*-scores) in the elderly (*n* = 619).

	Model 1			Model 2			Model 3			Model 3 PR by 100 g/d	
	β	SE	p *	β	SE	p *	β	SE	p *	PR **	95% CI
AWM											
Total dairy	0.01	0.02	0.62	0.01	0.02	0.80	0.02	0.02	0.78	0.98	0.88–1.09
Skimmed dairy	0.05	0.03	0.54	0.03	0.03	0.80	0.03	0.03	0.78	0.98	0.76–1.26
Semi-skimmed dairy	−0.02	0.03	0.66	−0.01	0.03	0.80	−0.01	0.03	0.78	0.92	0.72–1.17
Full-fat dairy	−0.04	0.04	0.91	0.00	0.04	0.96	0.01	0.04	0.78	0.85	0.59–1.24
Fermented dairy	0.03	0.03	0.62	0.02	0.03	0.80	0.01	0.03	0.78	0.92	0.72–1.17
Non-Fermented dairy	0.01	0.06	0.62	0.04	0.05	0.80	0.04	0.05	0.78	0.81	0.52–1.27
**EF**											
Total dairy	0.01	0.02	0.66	0.01	0.01	0.72	0.01	0.01	0.72	1.01	0.85–1.21
Skimmed dairy	0.07	0.02	0.01	0.05	0.02	0.06	0.05	0.02	0.06	0.96	0.73–1.26
Semi-skimmed dairy	−0.03	0.02	0.27	−0.02	0.02	0.54	−0.02	0.02	0.56	1.17	0.94–1.47
Full-fat dairy	−0.05	0.03	0.22	−0.02	0.03	0.68	−0.03	0.03	0.63	1.08	0.76–1.55
Fermented dairy	0.06	0.02	0.03	0.04	0.02	0.09	0.04	0.02	0.09	0.92	0.71–1.19
Non-Fermented dairy	−0.02	0.04	0.66	0.00	0.04	0.94	0.00	0.04	1.00	1.07	0.66–1.72
**IPS**											
Total dairy	−0.01	0.02	0.62	−0.01	0.02	0.56	−0.01	0.02	0.66	0.91	0.75–1.10
Skimmed dairy	0.06	0.03	0.12	0.04	0.02	0.36	0.04	0.02	0.36	0.87	0.65–1.15
Semi-skimmed dairy	−0.04	0.02	0.21	−0.04	0.02	0.36	−0.04	0.02	0.36	1.00	0.78–1.27
Full-fat dairy	−0.05	0.04	0.22	−0.03	0.04	0.56	−0.02	0.04	0.66	1.12	0.78–1.61
Fermented dairy	0.03	0.02	0.26	0.02	0.02	0.56	0.02	0.02	0.66	0.86	0.66–1.13
Non-Fermented dairy	−0.06	0.05	0.28	−0.03	0.05	0.56	−0.03	0.05	0.66	0.85	0.53–1.36
**EM**											
Total dairy	−0.02	0.02	0.68	−0.01	0.02	0.69	−0.01	0.02	0.57	1.08	0.92–1.27
Skimmed dairy	0.03	0.02	0.42	0.03	0.02	0.48	0.03	0.02	0.46	0.97	0.76–1.23
Semi-skimmed dairy	−0.04	0.02	0.42	−0.04	0.02	0.48	−0.04	0.02	0.46	1.18	0.95–1.45
Full-fat dairy	0.02	0.03	0.79	0.04	0.03	0.56	0.04	0.03	0.46	0.75	0.50–1.13
Fermented dairy	−0.01	0.02	0.79	−0.01	0.02	0.94	−0.01	0.02	0.92	1.09	0.87–1.37
Non-Fermented dairy	−0.01	0.05	0.79	−0.00	0.04	0.94	−0.01	0.04	0.92	0.99	0.63–1.57

Associations are adjusted for age, sex (Model 1), BMI, education, smoking, alcohol consumption, habitual physical activity (Model 2), total energy intake, dietary factors (Model 3). * Adjusted *p*-value using method by Benjami-Hochberg ** Total cheese and Dutch cheese by 30 g increase. AWM, attention and working memory; EF executive function; EM, episodic memory; IPS, information processing speed; PR, prevalence ratio.

**Table 4:** Associations between intake of individual dairy products (g/day) and cognitive performance (domain z-scores) in elderly (*n* = 619).

	Model 1			Model 2			Model 3			Model 3 PR by 100 g/d **	
	β	SE	p *	β	SE	p *	β	SE	p *	PR **	95% CI
AWM											
Total milk	−0.01	0.03	0.97	−0.01	0.03	0.94	−0.01	0.03	0.88	0.97	0.75–1.24
Semi/skimmed milk	−0.02	0.03	0.97	−0.01	0.03	0.94	−0.01	0.03	0.88	1.03	0.79–1.33
Full-fat milk	−0.05	0.08	0.97	−0.03	0.08	0.94	−0.03	0.08	0.88	1.74	0.52–1.74
Total yoghurt	0.02	0.04	0.97	0.01	0.04	0.94	0.02	0.04	0.88	0.79	0.54–1.14
Semi/skimmed yoghurt	0.04	0.04	0.97	0.03	0.04	0.94	0.03	0.04	0.88	0.72	0.48–1.09
Full-fat yoghurt	−0.02	0.08	0.97	−0.06	0.08	0.94	−0.05	0.08	0.88	0.91	0.35–1.81
Total cheese	−0.00	0.04	0.97	−0.03	0.04	0.94	−0.02	0.04	0.88	1.29	0.93–1.81
Dutch cheese	0.04	0.05	0.97	0.00	0.05	0.99	0.01	0.05	0.92	0.99	0.66–1.49
Chocolate milk	−0.01	0.07	0.97	0.04	0.06	0.94	0.05	0.06	0.88	0.76	0.41–1.39
Buttermilk	0.06	0.05	0.97	0.03	0.04	0.94	0.03	0.04	0.88	0.95	0.65–1.40
Custard	−0.08	0.10	0.97	0.06	0.09	0.94	0.06	0.09	0.88	0.87	0.39–1.93
**EF**											
Total milk	−0.02	0.06	0.39	−0.01	0.02	0.79	−0.02	0.02	0.68	1.15	0.90–1.47
Semi/skimmed milk	−0.03	0.02	0.40	−0.02	0.02	0.70	−0.03	0.02	0.72	1.21	0.94–1.56
Full-fat milk	−0.03	0.06	0.75	−0.02	0.06	0.81	−0.02	0.06	0.76	1.23	0.69–2.20
Total yoghurt	0.03	0.03	0.46	0.03	0.03	0.70	0.02	0.03	0.46	0.92	0.63–1.35
Semi/skimmed yoghurt	0.04	0.03	0.40	0.04	0.03	0.70	0.04	0.03	0.66	0.98	0.67–1.44
Full-fat yoghurt	0.03	0.06	0.75	−0.01	0.06	0.84	−0.02	0.06	0.76	1.09	0.51–2.31
Total cheese	0.01	0.03	0.76	−0.01	0.03	0.81	−0.02	0.03	0.72	0.78	0.51–1.18
Dutch cheese	0.07	0.04	0.33	0.03	0.04	0.70	0.02	0.04	0.72	0.95	0.65–1.40
Chocolate milk	−0.02	0.05	0.75	0.02	0.05	0.81	0.03	0.05	0.72	0.80	0.47–1.38
Buttermilk	0.10	0.03	0.03	0.08	0.03	0.19	0.08	0.03	0.19	0.99	0.66–1.50
Custard	−0.20	0.07	0.03	−0.09	0.07	0.70	−0.09	0.07	0.66	1.15	0.57–2.34
**IPS**											
Total milk	−0.02	0.03	0.67	−0.01	0.02	0.70	−0.01	0.03	0.84	1.01	0.75–1.34
Semi/skimmed milk	−0.04	0.03	0.33	−0.04	0.03	0.51	−0.04	0.03	0.44	1.04	0.79–1.36
Full-fat milk	0.05	0.07	0.67	0.05	0.07	0.70	0.05	0.07	0.77	1.09	0.56–2.12
Total yoghurt	−0.03	0.04	0.67	−0.04	0.03	0.70	−0.04	0.03	0.64	1.09	0.77–1.53
Semi/skimmed yoghurt	−0.01	0.04	0.87	−0.02	0.04	0.70	−0.02	0.04	0.83	1.07	0.75–1.53
Full-fat yoghurt	−0.08	0.07	0.48	−0.12	0.07	0.39	−0.12	0.07	0.39	1.43	0.71–2.89
Total cheese	0.02	0.04	0.75	−0.01	0.04	0.88	−0.00	0.04	0.96	0.80	0.54–1.18
Dutch cheese	0.09	0.04	0.26	0.05	0.04	0.55	0.05	0.04	0.61	0.67	0.47–0.97
Chocolate milk	−0.06	0.06	0.48	−0.03	0.05	0.70	−0.03	0.05	0.83	0.94	0.54–1.62
Buttermilk	0.11	0.04	0.11	0.08	0.04	0.33	0.08	0.04	0.33	0.57	0.31–1.06
Custard	−0.15	0.08	0.26	−0.04	0.08	0.70	−0.03	0.08	0.84	1.15	0.53–2.48
**EM**											
Total milk	0.00	0.02	0.98	0.00	0.02	0.97	0.00	0.03	0.97	1.03	0.79–1.35
Semi/skimmed milk	0.00	0.02	0.98	0.00	0.02	0.97	0.00	0.02	0.97	1.07	0.84–1.37
Full-fat milk	0.04	0.06	0.92	0.04	0.06	0.95	0.04	0.06	0.97	0.86	0.41–1.77
Total yoghurt	−0.04	0.03	0.80	−0.03	0.03	0.86	−0.03	0.03	0.86	1.22	0.89–1.69
Semi/skimmed yoghurt	−0.05	0.03	0.80	−0.04	0.03	0.83	−0.04	0.03	0,74	1.29	0.94–1.78
Full-fat yoghurt	0.07	0.06	0.80	0.06	0.06	0.83	0.07	0.06	0.74	0.74	0.35–1.53
Total cheese	−0.03	0.03	0.88	−0.04	0.03	0.83	−0.04	0.03	0.74	1.19	0.84–1.68
Dutch cheese	0.01	0.04	0.92	−0.01	0.04	0.97	−0.01	0.04	0.97	1.40	0.89–2.21
Chocolate milk	−0.04	0.05	0.88	−0.02	0.05	0.97	−0.02	0.05	0.97	0.87	0.50–1.51
Buttermilk	0.04	0.04	0.80	0.04	0.04	0.83	0.04	0.04	0.74	0.89	0.59–1.34
Custard	−0.02	0.08	0.92	0.02	0.08	0.97	0.03	0.08	0.70	0.48	0.18–1.29

Associations are adjusted for age, sex (Model 1), BMI, education, smoking, alcohol consumption, habitual physical activity (Model 2), total energy intake, dietary factors (Model 3). * Adjusted *p*-value using method by Benjamini–Hochberg ** Total cheese and Dutch cheese by 30 g increase. AWM, attention and working memory; EF executive function; EM, episodic memory; IPS, information processing speed; PR, prevalence ratio.
